# Tuberculosis burden caused by migrant population in Eastern China: evidence from notification records in Zhejiang Province during 2013–2017

**DOI:** 10.1186/s12879-022-07071-5

**Published:** 2022-01-31

**Authors:** Kui Liu, Songhua Chen, Yu Zhang, Tao Li, Bo Xie, Wei Wang, Fei Wang, Ying Peng, Liyun Ai, Bin Chen, Xiaomeng Wang, Jianmin Jiang

**Affiliations:** 1 Department of Tuberculosis Control and Prevention, Zhejiang Provincial Center for Disease Control and Prevention, Hangzhou, Zhejiang Province People’s Republic of China; 2 Key Laboratory of Vaccine, Prevention and Control of Infectious Disease of Zhejiang Province, Zhejiang Provincial Center for Disease Control and Prevention, Hangzhou, Zhejiang Province People’s Republic of China; 3 National Center for Tuberculosis Control and Prevention, China CDC, Beijing, People’s Republic of China; 4 School of Urban Design, Wuhan University, Wuhan, Hubei Province People’s Republic of China; 5 Hangzhou Center for Disease Control and Prevention, Hangzhou, Zhejiang Province People’s Republic of China

**Keywords:** Tuberculosis, Migrant population, Spatial–temporal analysis, Clustering

## Abstract

**Background:**

Internal migrants have an enormous impact on tuberculosis (TB) epidemic in China. Zhejiang Province, as one of the developed areas, also had a heavy burden caused by TB.

**Methods:**

In this study, we collected all cases in Zhejiang Province through the TB Management Information System from 2013 to 2017. Description analysis and Spatio-temporal analysis using R software and ArcGIS were performed to identify the epidemiological characteristics and clusterings, respectively.

**Results:**

48,756 individuals in total were notified with TB among the migrant population (TBMP), accounting for one-third of all cases identified. The primary sources of TB from migrants outside the province were from Guizhou, Sichuan, and Anhui. Wenzhou, Taizhou, and Lishui were the three mainly outflowing cities among the intra-provincial TBMP and Hangzhou as the primarily inflowing city. Also, results implied that the inconsistency of the TBMP in spatial analysis and the border area of Quzhou and Lishui city had the highest risk of TB occurrence among the migrants. Additionally, one most likely cluster and four secondary clusters were identified by the spatial–temporal analysis.

**Conclusion:**

The effective control of TB in extra-provincial MP was critical to lowering the TB burden of MP in Zhejiang Province. Also, it is suggested that active TB screening for migrant employees outflowed from high epidemic regions should be strengthened, and further traceability analysis needs to be investigated to clarify the mechanism of TB transmission in clustered areas.

**Supplementary Information:**

The online version contains supplementary material available at 10.1186/s12879-022-07071-5.

## Introduction

Tuberculosis (TB) had aroused a considerable disease burden globally, which caused 10 million people falling ill only in 2018 [1]. As one of the high epidemic countries, China accounted for 9% of the global total and 14% of worldwide disease burden [2]. Although increasing countries had performed several endeavors in prevention, diagnosis, and treatment to accelerate the TB responses, available speed was far below the requirement of End TB [2, 3]. Migration flows consisted of intra-provincial and extra-provincial flows, as one of the adverse influencing factors for TB control, caused a massive challenge for the Stop TB strategy [4]. In China, people from underdeveloped areas commonly moved to the developed provinces that owned more opportunities for employment and outstanding income, which accounted for 20% of the whole population [5]. The migrant population (MP) brought significant economic development to the inflow area and simultaneously created unprecedented challenges and issues such as transportation system, education resources, and health accessibility [6, 7]. For these specific groups, adverse factors for TB occurrence like limited living conditions and low economic levels were still existing [8, 9]. Thus, some developed areas such as eastern coastal provinces in China confront tremendous pressure and challenge for TB control in MP.

The spatial–temporal analysis had been widely used in communicable diseases to determine the potential clusterings, which had its potential merits than conventional epidemiology methods [10]. It could determine the cluster patterns of epidemics in multiply dimensions involving time, space, and the combination of both. The results from spatial–temporal analysis in infectious diseases might contribute to rapidly controlling the epidemics, realizing the health policy precisely, and optimally promoting health resources.

Zhejiang Province has a medium TB burden with the massive MP in China [11–14]. This study aimed to explore the potential influence of MP on the control and prevention of TB in Zhejiang Province. We additionally investigated how TBMP spatially moved in the province during the study time.

## Methods

### Overview of the study area

Zhejiang Province is located in the eastern region of China, covering two sub-provincial cities like Hangzhou and Ningbo and nine other regional cities (specifically, Wenzhou, Jiaxing, Huzhou, Shaoxing, Jinhua, Quzhou, Zhoushan, Taizhou, and Lishui) [10]. The number of permanent residents was 58.50 million in 2019, along with approximately 26 million migrants ranked second nationally in 2018 [13, 14].

### Data collection of TBMP cases

Screened and omitted the personal items, all notified TB cases in Zhejiang Province were extracted from the TB information management system between 2013 and 2017 [15]. All notified TB cases consisted of confirmed pulmonary TB (PTB), clinical diagnostic PTB, and extrapulmonary TB (EPTB) as well, which referred to National Diagnostic Criteria for Pulmonary Tuberculosis (WS288–2008 and WS 288-2017) and Classification of Tuberculosis (WS196-2017) [16, 17]. The definition for TB diagnostic classifications was introduced in our previous study [18]. For each case record, the information of sex, age, year, occupation, registration address (county-level), current address code (CAC), and permanent address code (PAC), etc., was retained in our study. Then, the matching analysis of CAC and PAC was performed to decide the source of all participants. The six digital address codes of both CAC and PAC orderly represented the province, the city, and the county. Therefore, if the first two codes of CAC represented Zhejiang Province while the PAC was not, the case was defined as the extra-provincial MP (E-PMP); the first two codes of CAC and PAC represented Zhejiang Province while the middle two codes were different, the case was denoted as extra-urban MP (E-UMP). By that analogy, the last two codes of CAC and PAC determined whether the case belonged to the intra-urban MP (I-UMP) or not. All MP data was collected from the statistics department of the health sector.

Characteristics of migrant patients were collected, including notified TB incidence, ethnicity, gender, and the top three occupations of variable subgroups. The main flow features of notified cases in both extra-province and intra-province were shown to identify the primary sources of notified TBMP.

### Statistical analysis

The distributional characteristics of all TBMP in Zhejiang Province were exhibited by county-level during 2013–2017. Meanwhile, the risk map of TBMP was shown by the method of inverse distance weighted (IDW) interpolation [19, 20]. To detect the geographical anomalies in TBMP, the trend face analysis was used to present the notified TBMP incidence, in which x and y represented different directions, and z denoted the notified incidence [21, 22]. Additionally, the spatial analysis was carried out by both general spatial autocorrelation analysis and local spatial autocorrelation analysis [23]. The general autocorrelation used the Global Moran's I Index:

$${\text{I}} = \frac{n}{{\mathop \sum \nolimits_{i = 1}^{n} \mathop \sum \nolimits_{j = 1}^{n} w_{i,j} }}\frac{{\mathop \sum \nolimits_{i = 1}^{n} \mathop \sum \nolimits_{j = 1}^{n} w_{i,j} \left( {X_{i} - \overline{X}} \right)\left( {X_{j} - \overline{X}} \right)}}{{\mathop \sum \nolimits_{i = 1}^{n} \left( {X_{i} - \overline{X}} \right)^{2} }}$$

In this Index, n represented the number of counties in Zhejiang Province, ${X}_{i}$ and ${X}_{j}$ represented autocorrelations from county *i* and *j*, *w*_*i,j*_ was the matrix of spatial weights. If county *i* was adjoined to county *j*, ${w}_{i,j}=1$, otherwise ${w}_{i,j}=0$. The range of Moran's I Index ranged from − 1 to 1. If Moran's Index was greater than zero, it implied possible clustering in the spatial distribution (also called positive association). If Moran's Index was below zero, the result was deemed as dispersing in the spatial distribution (also negative association); if Moran's Index = 0, it meant a random spatial distribution [24, 25]. In the meantime, Z-scores were used to test the significance of spatial aggregation or statistical clustering. If Z-scores were above or equal to 1.96 or *P* values were below 0.05, the spatial pattern demonstrated a potential relevance [11]. Local Getis-Ord G was employed to locate hot or cold spots and was estimated by the Gi* value [26, 27].

To conduct spatial–temporal analyses, we used Kulldorff's space–time scan statistics to identify the clusterings in specific combinations of time and location [28]. The Kulldorff method uses a moving cylindrical window to scan the region of the study, in which the height of the cylinder represented the possible clustering time, and the base of the cylinder denoted the clustered areas [29]. Both the maximum cluster of spatial and temporal size set to 50%. Log-likelihood ratios were calculated to identify clusters by comparing the observed and expected number of migrants with TB. The significance of the identified cluster at a 95% confidence level was examined using Monte Carlo Simulations [29, 30]. Descriptive analyses were performed by R software (version 3.5.3). ArcGIS software (version 10.2, SERI Inc.; Redlands, CA, USA) and SaTScan (version 9.1.1, Boston, MA, USA) were used to depict spatial and spatial–temporal results.

## Results

### Characteristics of notified TB cases among migrants

From 2013 to 2017 in Zhejiang Province, a total of 153,914 TB cases were notified, of which 48,756 (31.7%) were among migrants. Among the TBMP, it was classified into extra-provincial TBMP (E-PTBMP, 44,377, accounted for 91%) and intro-provincial TBMP (I-PTBMP, 4379, accounted for 9%). The sex ratio of males and females in TBMP cases was 2:1. The TBMP notification rate continuously declined throughout the study period starting at 44.5 cases per 100 thousand persons in 2013, 46.0 cases per 100 thousand persons in 2014, 40.5 cases per 100 thousand persons in 2015, 36.5 cases per 100 thousand persons in 2016, and 36.6 cases per 100 thousand persons in 2017, respectively (*P* for trend = 0.029). The decline trend was greater than that in general population. The top 5 ethnic groups in TBMP were Han (90.8%), Miao (2.4%), Tujia (1.7%), Yi (1.4%), and Bouyei (1.3%) during the study period. Furthermore, the I-PTBMP was classified into the intra-urban TBMP (I-UTBMP, 1653 cases) and extra-urban TBMP (E-UTBMP, 2726 cases). The top 3 occupations of notified TBMP cases were listed in Table 1.

**Table 1:** The Top Three Occupations of Notified Tuberculosis among the Migrant Population from 2013 to 2017

	Year					
	2013	2014	2015	2016	2017	Total (%)
**E-PTBMP**						
Migrant worker	3847	3245	2427	2144	2105	13,768 (31.0)
Peasant	1617	1918	1891	2036	2111	9573 (21.6)
Household service	1247	1634	1548	1335	1464	7228 (16.3)
**E-UTBMP**						
Peasant	84	107	113	110	145	559 (20.5)
Household service	73	73	76	92	65	379 (13.9)
Student	64	85	69	76	53	347 (12.7)
**I-UTBMP**						
Peasant	42	46	82	92	128	390 (23.6)
Worker	35	43	60	57	56	251 (15.2)
Household service	31	39	34	52	57	213 (12.9)

E-PTBMP: extra-provincial TB cases among migrant population, E-UTBMP: extra-urban TB cases among migrant population, I-UTBMP: intra-urban TB cases among migrant population

**Fig. 1:**
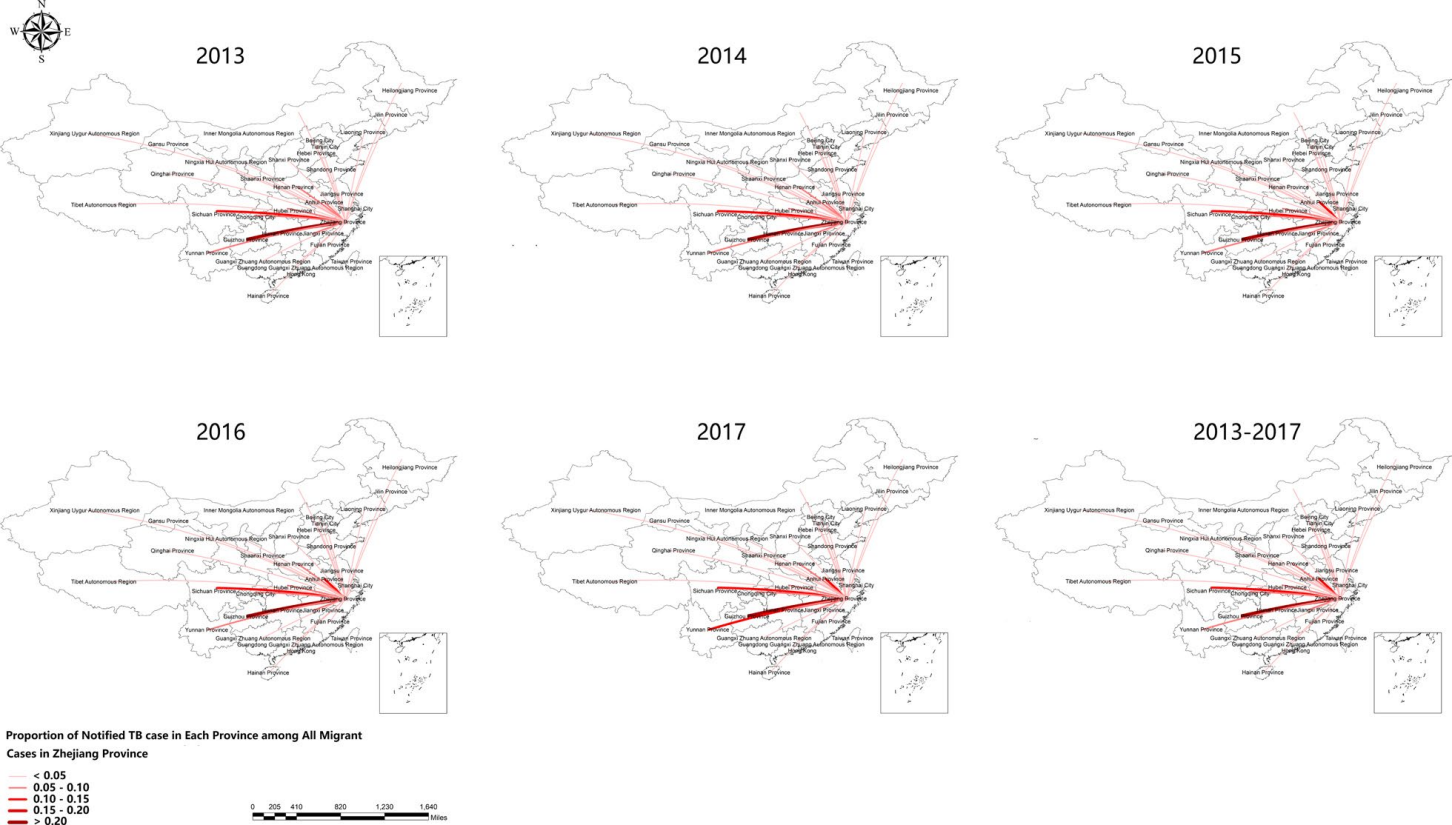
The Movement of Notified TB Cases from Extra-provincial MP in Zhejiang Province during the Study Period. The red line represented the current tuberculosis cases flowing from other provinces to Zhejiang Province. The line width and shade showed the proportion of all tuberculosis migrants. These were created by ArcGIS software (version 10.2, ESRI Inc.; Redlands, CA, USA); URL https://www.esri.com/

**Fig. 2:**
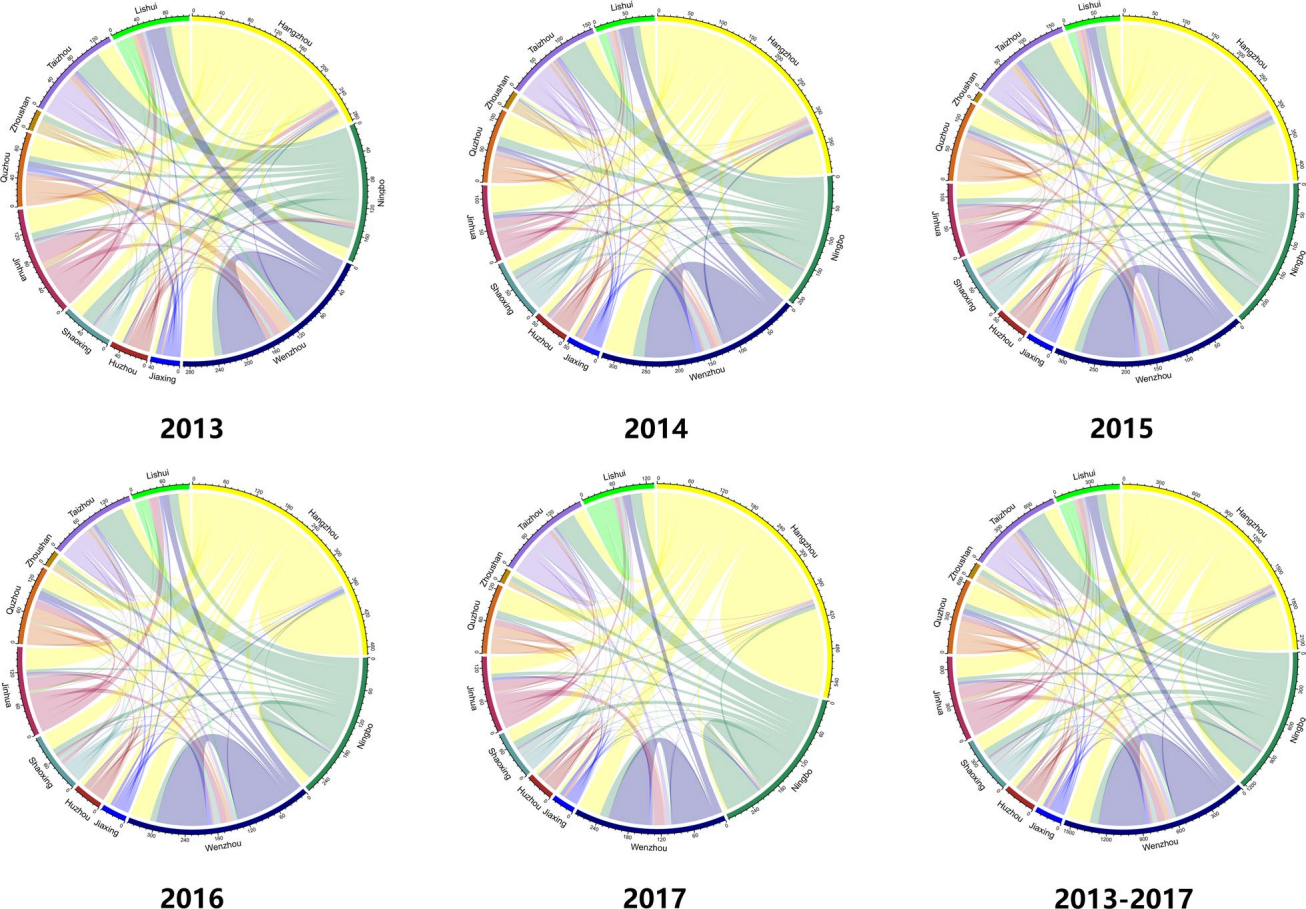
The Mobility of Notified TB Cases among the Intra-urban MP and Extra-urban MP in Zhejiang Province. The side length and its scale on the outside of the circle represent the number of TBMP from the interior of Zhejiang Province, including both Intra-urban TBMP and Extra-urban TBMP; the direction of variable colors inside the circle represents its origin. These were created by R software (3.5.3); URL http://www.rproject.org/

All included TBMP were categorized by treatment, registration, diagnosis, and etiology status. The details were shown in Table 2. Besides, the characteristics of notified TBMP were analyzed, and available results displayed that Guizhou Province (23%), Sichuan Province (10.5%), and Anhui Province (9.4%), ranked the top 3, were the primary source of notified TB cases in E-PMP, which was revealed in Fig. 1. Among E-UTBMP in Zhejiang Province, notified cases from Wenzhou, Taizhou, and Lishui city were the top 3 sources, accounting for 19.6%, 16.9%, and 14.9%, while the main in-flowing region was Hangzhou city. Additionally, for I-UTBMP, Hangzhou, Wenzhou, and Ningbo city existed the major internal flow of notified cases (Additional file 1: Table S1). This information was presented in Fig. 2.

**Table 2:** The Epidemiology Classification of Diagnosed Tuberculosis among Migrant Population in Zhejiang Province during the Study Period

	Year					Total (%)
	2013	2014	2015	2016	2017	
**Treatment classification**						
Initial treatment	9490	9630	8689	8524	8488	44,821 (91.9)
Retreatment	844	858	754	710	769	3,935 (8.1)
**Registration classification**						
New cases	9490	9630	8689	8524	8488	44,821 (91.9)
Relapse cases	640	634	508	543	561	2,886 (5.9)
Others	204	224	246	167	208	1,049 (2.2)
**Diagnosis classification**						
Primary tuberculosis	3	5	3	4	2	17 (< 0.1)
Hematogenous disseminated tuberculosis	74	59	46	48	46	273 (0.6)
Secondary tuberculosis	9451	9635	8742	8478	8439	44,745 (91.8)
Tuberculous pleuritis	659	616	526	576	624	3,001 (6.2)
Extra pulmonary tuberculosis	147	173	126	128	146	720 (1.5)
**Etiological classification**						
Positive^a^	3495	3382	2821	2677	2894	15,269 (31.3)
Negative or unknown	6839	7106	6622	6557	6363	33,487 (68.7)

^a^It consisted of positive sputum smear, positive sputum culture, positive rapid molecular diagnosis, and positive etiology

### Spatial distribution of notified TBMP in Zhejiang Province

**Fig. 3:**
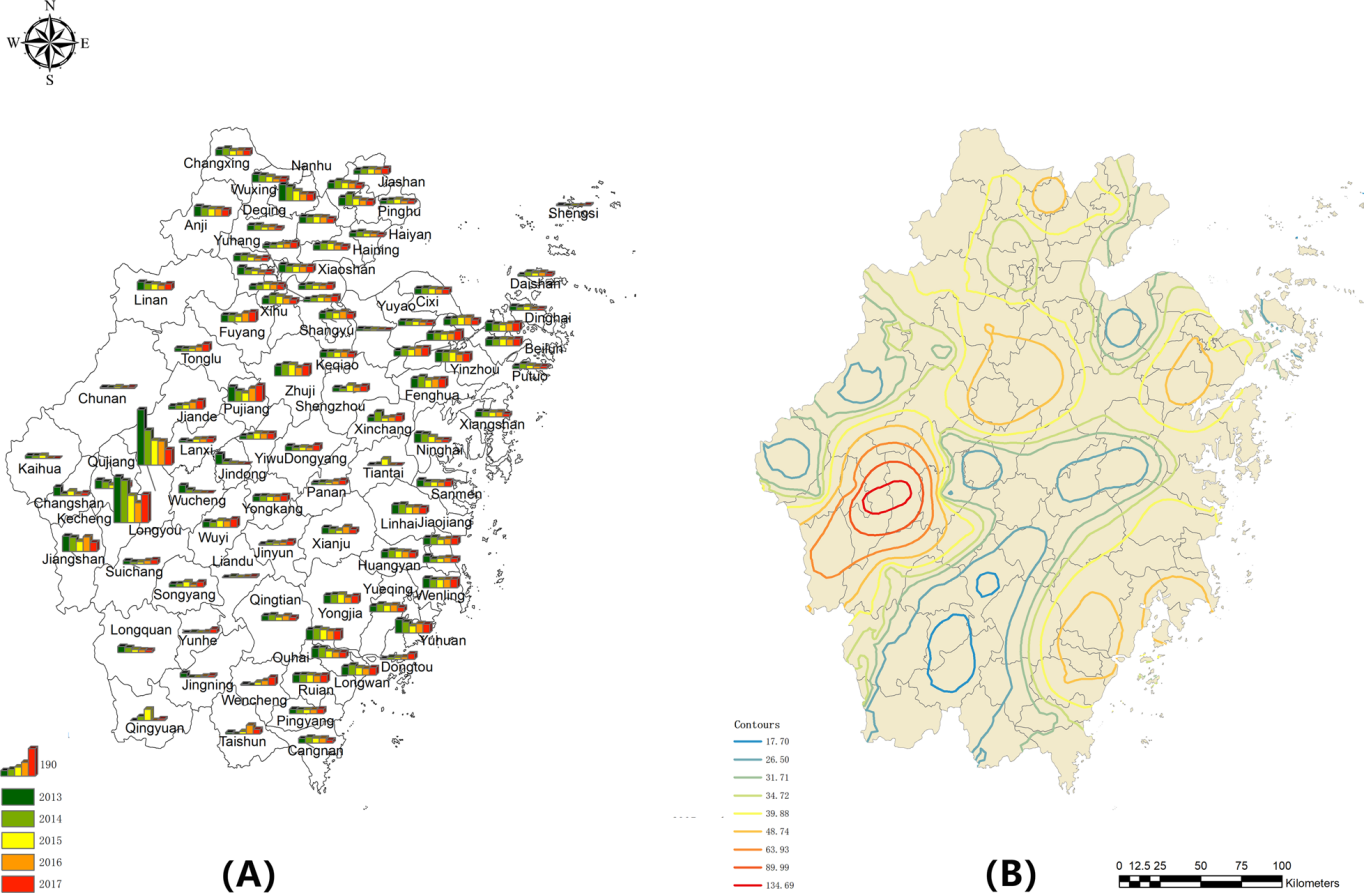
Map of Notified TB Incidence among MP (**A**) and its Prediction Map by IDW (**B**). **A** The height of the five columns in each region represents notification incidence and year; **B** Different colored circles represent different risk layers, with red having the highest risk and blue having the lowest risk. These were created by ArcGIS software (version 10.2, ESRI Inc.; Redlands, CA, USA); URL https://www.esri.com/

From 2013 to 2017, the notified incidences of TBMP in Zhejiang Province were exhibited by county level in Fig. 3A, and the IDW interpolation was performed to show the prediction of the risk map in Fig. 3B. The higher notified TB incidence among MP was found in the southwest of Zhejiang Province while other regions were relatively stable. Besides, during the study period, the further trend surface analysis implied that the notified TB incidence in MP (Z) showed a decreased trend in the direction of West to East (X) and South-North (Y) in Additional file 1: Fig. S1(A). From the other orientation of West–East to South-North (X) and Southwest-Northeast to Southeast-Northwest (Y) in Additional file 1: Fig. S1(B), it demonstrated an inversed U trend and an increasing trend, respectively.

### Spatial analyses among TBMP

**Fig. 4:**
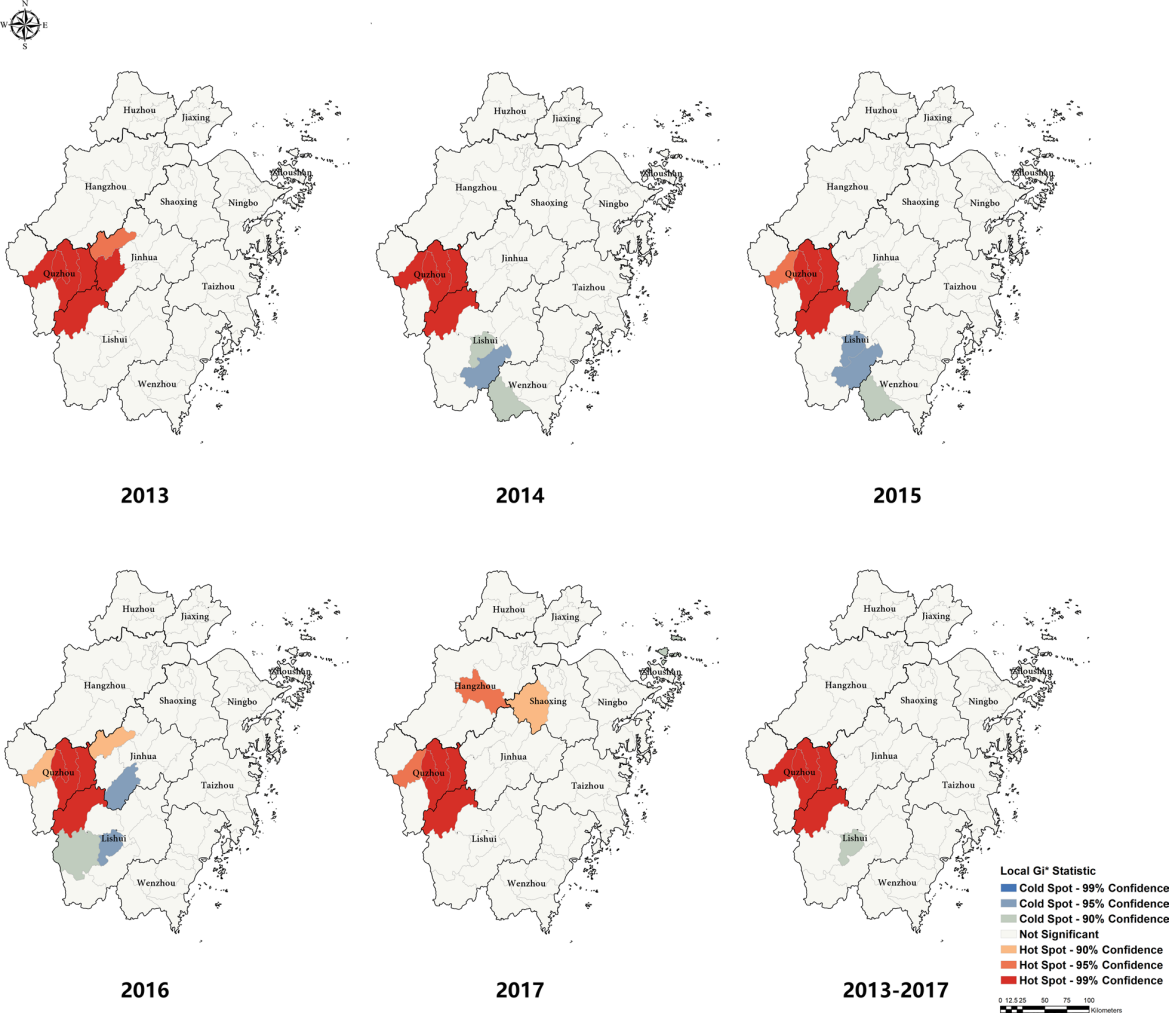
Local Getis's Gi Results of Hot Spot and Cold Spot for Notified TB Incidence among MP in Zhejiang Province. The local Getis's Gi had identified the hot spot and cold spot with different colors. Hot spot implied the potential clusters of TB epidemic in migrants, and cold spot hinted low TB risk among MP in these regions. These were created by ArcGIS software (version 10.2, ESRI Inc.; Redlands, CA, USA); URL https://www.esri.com/

For the result of general spatial autocorrelation, there was significant spatial heterogeneity in 2013 and 2014, while there were no statistically significant  differences in 2015–2017 (Table 3). In Fig. 4, we classified the clusterings with three levels at 90%, 95%, and 99% confidence based on the test value of Gi* Z-score. In 2013, hot spots among the MP were clustered in the western regions of the province. Hot spots were mainly distributed in the cross of some counties in Lishui and Quzhou city like Longyou, Qujiang, Kecheng, Changshan, and Suichang. For the cold spots, the results were not steady during the study period. For instance, the cold spots located in Lishui and Wenzhou city junction during 2014–2015 appeared in Jinhua and Lishui city in 2016.

**Table 3:** General Spatial Autocorrelation Analysis of Notified TB Incidence among MP in Zhejiang Province during the Study Period

Year	Moran’s I index	Z-score	*P*-value
2013	0.196	3.367	0.001
2014	0.152	2.522	0.012
2015	0.109	1.789	0.074
2016	0.120	1.884	0.060
2017	0.120	1.945	0.052
2013–2017	0.138	2.279	0.023

**Fig. 5:**
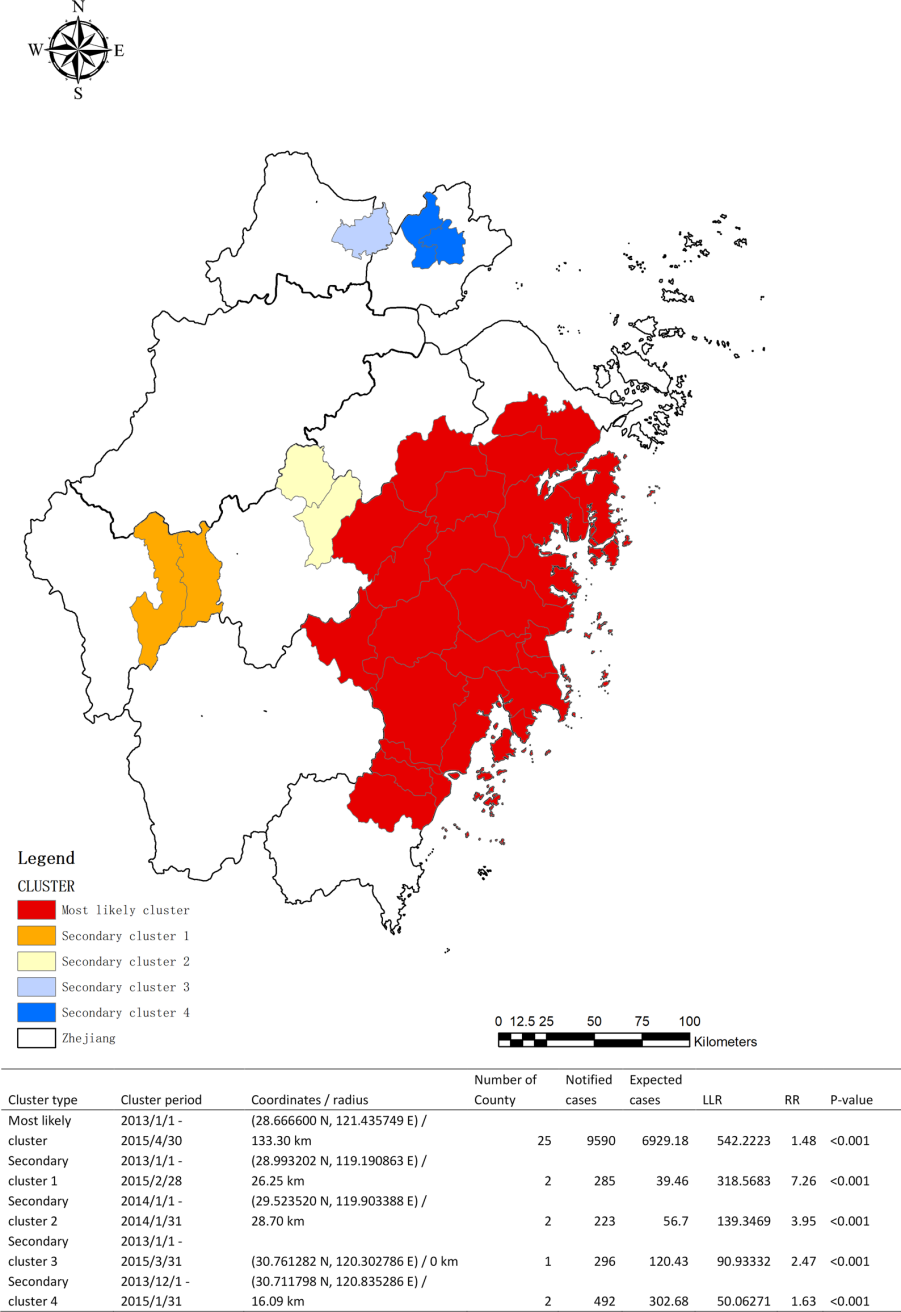
Spatial–temporal Clustering of Notified TBMP from 2013 to 2017 in Zhejiang Province. This map showed one most likely cluster and four secondary clusters with different time dimensions during the study period. The red regions showed the most likely cluster with 25 counties and an RR value of 1.48. This map was created by ArcGIS software with the Homepage of https://www.esri.com/ (version 10.1, ESRI Inc.; Redlands, CA, USA) and SaTScan software (version 9.1.1, Boston, MA, USA). SaTScan TM is a trademark of Martin Kulldorff. The SaTScan TM software was developed under the joint auspices of (i) Martin Kulldorff, (ii) the National Cancer Institute, and (iii) Farzad Mostashari of the New York City Department of Health and Mental Hygiene

We found one most likely cluster and four secondary clusters by Kulldorff's space–time scan statistics. For the most likely cluster, it consisted of 25 counties in the eastern region of Zhejiang Province with 9590 notified TB cases among migrants, in which the cluster period was from January 2013 to April 2015. The risk ratio in this cluster was 1.48 times higher than in other regions. Other secondary clusterings were presented in Fig. 5.

## Discussion

To our knowledge, available literature showed that the prevention and control of active TB in MP meant an enormous challenge globally. MP typically has lower awareness of TB, causing potential delay diagnosis [31–33]. Besides, Non-adherence to anti-TB treatment is common among MP, which may increase the risk of TB transmission and the development of drug resistance [34]. Thus, in the context of substantial population movements, identification and understanding of its epidemiology features in diverse dimensions would help reduce TB's burden in the eastern areas of China.

We found that diagnosed migrant TB accounted for nearly one-third of all cases during the study period. Additionally, among TBMP, the number of TB in E-PMP is greater than that in I-PMP nearly ten times. Therefore, the effective control of TB in E-PMP was critical to lowering the TBMP burden in Zhejiang Province. Also, it showed a consistent decline in diagnosed TB incidence among both migrants and general groups, accompanied by a more considerable fall in the former. According to previous studies, several possible reasons may explain the phenomenon above, including the continuously aging population among permanent residents and the overwhelming majority of migrants under 40 years old [35, 36]. Furthermore, Zhejiang Province carried out the global fund project from 2006 to 2012 in MP. The comprehensive implementations were put into practice for controlling TBMP, including the supply of transportation and nutrition benefits, the extension for outpatient service, etc*.*, greatly improved treatment success in this group. After that, although the project was ended up, it still caused the successive decline of TB notification rate in both MP and the general population due to several successful experiences that were retained and continued. Further attentions to control TBMP should be paid to integrate prevention and control strategies systematically, constituting improving early detection, advancing community management, and constructing the long-term mechanism in policy development.

Our findings showed that notified TB cases among MP were mainly from Guizhou, Sichuan, and Anhui Province. Based on the national surveillance report in 2018, the notified TB incidence in these provinces was higher than that in Zhejiang Province, even 2.56 times in Guizhou Province. Thus, performing active screening or regular physical examination for employees from high epidemic regions should be considered and strengthened; due to costly hospitalizations and no available insurance, an eligible study proved that the catastrophic costs of TB care might further lead to the unwillingness to treatment-seeking and the subsequent interruption in TB treatment in these specific groups [37]. In Zhejiang Province, the reimbursement ratio in permanent residents claimed to be no less than 70% for ordinary TB cases and 90% for drug-resistant TB cases [38]. Hence, it is suggested that a sharing mechanism of cross-provincial health insurance making MP enjoy the same policies as local should be considered.

In our research, Wenzhou, Taizhou, and Lishui were the top three mainly outflowing cities, while the primarily inflowing city was Hangzhou. As the capital of Zhejiang Province, Hangzhou owned more employment opportunities and developed market economy that attracted more MP. In addition, as the top 3 GDP cities in Zhejiang Province, Hangzhou, Wenzhou, and Ningbo city showed the maximum three intra-urban mobility, which also implied the impact by the economic level. Thus, for community management, TB health education for MP in these developed regions should be promoted. Also, combined with community doctors or clinical TB specialists, the gratuitous treatment activity on the weekend could improve the early identification and diagnosis of active TB in MP. Moreover, some convenient measures such as the "Green Channel" also should be considered to set for TB among MP in designated hospitals to offset the potential disequilibrium in these groups.

In the spatial analysis, eligible results implied the inconsistency of the TB risk among MP across the province, demonstrating an existing spatial heterogeneity during the study period. The western region of Zhejiang Province, especially the border area of Quzhou and Lishui city, had the highest risk. Previous studies also suggested that the Lishui and Quzhou cities had higher TB epidemics than others [11]. Although available evidence in this study might not identify the causal relationship between TB epidemics in local and that in MP or the complex interaction of both, traceability analysis of Mycobacterium TB in the ongoing future should be designed to clarify the potential mechanism of TB transmission in these regions, ultimately reducing the TB occurrence.

Spatio-temporal analysis showed that the combined clusters of TB epidemics in MP were mainly concentrated in the first three years, and the most prominent clustering located in the southeast coastal region resulted from the developed economy attracted more MP. After that, the apparent clusters of TB epidemics in MP were not observed. According to the Chinese Development Report among MP in 2018, the scale of MP has changed from a previously continuous rise to a slow decline since 2015 [39]. That is to say, as a principal labor-importing province, the disappearance of TB clusters might be in correlation with the decreased trend of the MP scale. Meanwhile, the industrial upgrading and strengthening of management might promote the health examination in the workplace that reduces the occurrence of TB clustering in MP.

Several limitations to this study should be mentioned. We used a matching method to identify migrants and permanent residents based on permanent and current address codes. Misclassification of prior migrants may occur if household information is inaccurate or not updated. Secondly, the administrative region of some counties in Zhejiang Province had changed in our study period. To avoid this influence, we counted the notified TB incidence of all neighbor counties with change as an entirety, which might ignore the difference in various regions to some extent.

## Conclusion

In conclusion, we evaluated the TB epidemiological characteristics among MP in Zhejiang Province, which helped identify the principal source and develop the appropriate implementations or policies to control the total TB epidemics in Zhejiang Province. For MP, it implied that the effective control of TB in E-PMP was the critical point to lower the TB burden of MP in Zhejiang Province. Meanwhile, some implementations such as performing active screening or regular physician examination for employees from high epidemics regions should be strengthened. Ultimately, the border area of Quzhou and Lishui city should strengthen the management and control of the TB epidemic among MP, and further traceability analysis needs to be investigated and explored to clarify the mechanism of TB transmission in clustered areas.

## Supplementary Information

**Additional file 1: Table S1.** The Notified Intra-urban TBMP in Zhejiang Province during the Study Period. **Figure S1.** Trend Surface Analysis of Notified TB Incidence among MP during 2013-2017. (A) X for the direction of West to East, Y for South to North, and Z represented the notified TB incidence in MP. (B) X for the direction of West-East to South-North, Y for Southwest-Northeast to Southeast-Northwest, and Z represented the notified TB incidence in MP.

## Data Availability

All data and materials were included in this paper. The corresponding author could provide all data upon reasonable request.

## Abbreviations

TB: Tuberculosis
TBMP: TB among the migrant population
MP: Migrant population
PTB: Pulmonary TB
EPTB: Extrapulmonary TB
CAC: Current address code
PAC: Permanent address code
E-PMP: Extra-provincial MP
E-UMP: Extra-urban MP
I-UMP: Intra-urban MP
IDW: Inverse distance weighted
E-PTBMP: Extra-provincial TBMP
I-PTBMP: Intra-provincial TBMP
I-UTBMP: Intra-urban TBMP
E-UTBMP: Extra-urban TBMP
