# Supplementary material for: Tuberculosis burden caused by migrant population in Eastern China: evidence from notification records in Zhejiang Province during 2013–2017
Source: BMC Infect Dis. 2022 Jan 31;22:109. doi: 10.1186/s12879-022-07071-5 (PMC8805310; doi:10.1186/s12879-022-07071-5)

Supplement table and figure’s legend

sTable 1. The Notified Intra-urban TBMP in Zhejiang Province during the Study Period.

|  |  |  | Year |  |  |  |
| --- | --- | --- | --- | --- | --- | --- |
| City | 2013 | 2014 | 2015 | 2016 | 2017 | Total |
| Hangzhou City | 18 | 67 | 100 | 121 | 168 | 474 |
| Wenzhou City | 67 | 85 | 93 | 100 | 79 | 424 |
| Ningbo City | 29 | 36 | 41 | 61 | 69 | 236 |
| Taizhou City | 25 | 21 | 18 | 25 | 38 | 127 |
| Jinhua City | 28 | 13 | 18 | 24 | 19 | 102 |
| Quzhou City | 4 | 14 | 22 | 27 | 21 | 88 |
| Lishui City | 10 | 6 | 5 | 10 | 26 | 57 |
| Shaoxing City | 4 | 14 | 14 | 10 | 12 | 54 |
| Huzhou City | 4 | 4 | 10 | 11 | 10 | 39 |
| Jiaxing City | 6 | 3 | 8 | 5 | 6 | 28 |
| Zhoushan City | 6 | 8 | 1 | 5 | 4 | 24 |

sFigure 1. Trend Surface Analysis of Notified TB Incidence among MP during 2013-2017.

(A) X for the direction of West to East, Y for South to North, and Z represented the notified TB incidence in MP.

(B) X for the direction of West-East to South-North, Y for Southwest-Northeast to Southeast-Northwest, and Z represented the notified TB incidence in MP.


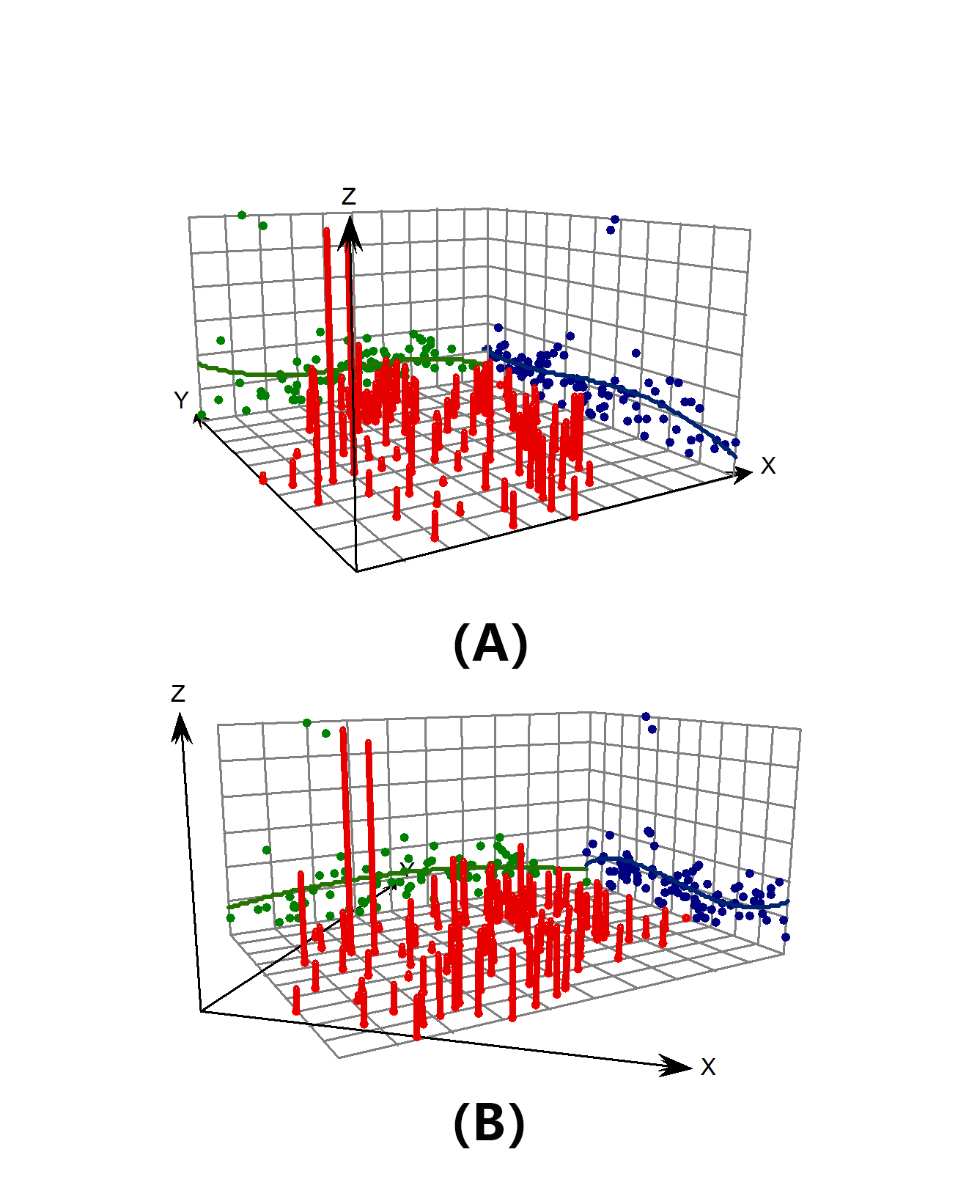

Supplement: Supplementary file 1 — Additional file 1: Table S1. The Notified Intra-urban TBMP in Zhejiang Province during the Study Period. Figure S1. Trend Surface Analysis of Notified TB Incidence among MP during 2013-2017. (A) X for the direction of West to East, Y for South to North, and Z represented the notified TB incidence in MP. (B) X for the direction of West-East to South-North, Y for Southwest-Northeast to Southeast-Northwest, and Z represented the notified TB incidence in MP. [file 12879_2022_7071_MOESM1_ESM.docx]
